# Research trend and hotspots of polycystic ovary syndrome with depression from 1993 to 2024: a bibliometric analysis

**DOI:** 10.3389/fgwh.2024.1468471

**Published:** 2024-11-28

**Authors:** Jing Xie, Yi Cao, Qian Wen, Xuxia Song, Yuanyuan Shi, Xia Gao

**Affiliations:** ^1^ Biomedical Center, Qingdao University, Qingdao, Shandong, China; ^2^ Department of Acupuncture, Qingdao Central Hospital, University of Health and Rehabilitation Sciences, Qingdao, Shandong, China

**Keywords:** polycystic ovary syndrome (PCOS), depression, bibliometrics, quality of life, oxidative stress, inflammation, gut microbiota, obstructive sleep apnea

## Abstract

**Background:**

Polycystic ovary syndrome (PCOS), a common endocrine disorder, affects women of reproductive age, and its adverse consequences affect women throughout their lifespan, from adolescence to postmenopause. The prevalence of depression is much higher in women with PCOS than in healthy controls. Thus, it is recommended that depressive syndrome be screened routinely in all patients with PCOS at diagnosis. To date, no comprehensive bibliometric analysis has been conducted in this field. Therefore, we conducted a bibliometric analysis to describe the current status, trends, and hotspots of PCOS research related to depression.

**Materials and methods:**

Using data retrieved from the Web of Science (WoS) Core Collection database from 1993 to 2024, bibliometric analyses were performed using WoS and CiteSpace software.

**Results:**

Since the first paper was published in 1993, studies related to PCOS and depression have remained rare in the following decade. Since the establishment of the Rotterdam criteria in 2003, research on the etiology, pathogenesis, and treatment of PCOS with depressive syndrome has entered a booming period. The United States and Australia indisputably took leading positions in this area, with the most outstanding institutions in the world being the University of Pennsylvania and Monash University. Although achievements have flourished since 2003, the exact pathogenesis of PCOS remains uncertain owing to its heterogeneity. New research is rapidly increasing to fill these gaps and to push forward the goal of improving the quality of life in women with PCOS and depression. Along with progress in research, the world's leading societies organize conferences every 5 years to update guidelines for the assessment and management of PCOS. “Oxidative stress,” “inflammation,” “obstructive sleep apnea,” “gut microbiota,” and “single nucleotide polymorphism” appeared as new hotspots in the recent 5 years.

**Conclusion:**

A bibliometric analysis was performed to describe the trends and hotspots of research in women with PCOS and depression to attract the attention of more researchers to this topic.

**Systematic Review Registration:**

https://www.webofscience.com/wos/woscc/basic-search.

## 1 Introduction

Polycystic ovary syndrome (PCOS), the most common endocrine disorder, affects 8 to 13% women of reproductive age (1). The International Evidence-based Guidelines in 2018 endorsed the diagnostic criteria for PCOS as (i) clinical or biochemical hyperandrogenism, (ii) oligo- or anovulation, and (iii) polycystic ovaries on ultrasound, which are recommended to be replaced by the anti-Müllerian hormone in 2023 (2). When (i) and (ii) are present, condition (iii) is not necessary for diagnosis.

Depression was ranked as the third major cause of disease burden globally by the World Health Organization in 2008, and it is estimated that the disease will rank first by 2030 (3, 4). Symptoms of depression increase significantly in patients with PCOS, with a prevalence rate of 25.7%, which is three to eight times higher in women with PCOS than in controls (5–7). Owing to the association between PCOS and depression, depressive syndrome is recommended in the guidelines to be screened routinely in all patients with PCOS at diagnosis with psychological assessment and therapy, as indicated (8).

Possible shared links have been described between PCOS and depression, e.g., stress-related abnormal HPA axis, obesity, insulin resistance (IR), androgen excess, infertility, inflammation, insulin-like growth factor 1, vitamin D and hypofibrinolysis, although further validation is still needed for these shared links (6, 9–13). These possible links not only induce the pathogenesis of both PCOS and depression but also form a complicated interplay network, and patients with PCOS experience a vicious cycle in the network under the shadow of depression. For example, prolonged exposure to stress leads to menstrual irregularities and reproductive disorders, resulting in infertility, a characteristic of PCOS. However, stress and infertility, as well as other psychiatric burdens, such as an unsatisfactory image of obesity and androgen excess-induced acne, exacerbate depression. Depression is accompanied by stress-related hyperactivity of the HPA axis; hypercortisolism can induce obesity and IR. In turn, IR leads to hyperinsulinemia, which causes excessive production of androgens in the ovary, another hallmark of PCOS and one of the reasons for infertility. Levels of cytokines including tumor necrosis factor-ɑ, interleukin (IL)-1, and IL-6 are greatly increased in women with PCOS. These peripheral pro-inflammatory cytokines produced by the gut microbiota or fat tissue in patients with PCOS may cross the blood–brain barrier to affect the release of neurotransmitters, e.g., 5-HT and dopamine, leading to depression. Obesity is a common comorbidity in 80% of patients that can lead to depression and vice versa (10). The adipose tissue in obese women is a source of multiple inflammatory cytokines that are involved in the pathogenesis of PCOS and depression. Hypofibrinolysis, mainly caused by an increase of plasminogen activator inhibitor type 1 (PAI-1), is considered as an important element in both of the pathogenesis of PCOS and depression in recent years. The increased levels of PAI-1, induced by some pro-inflammatory cytokines, glucose, insulin, and cortisol, are reported to associate with PCOS as well as depression (13–15).

Several studies have investigated the association between PCOS and depression. Bibliometrics has emerged as a method for analyzing published literature quantitatively and evaluating trends in certain research fields based on statistical techniques (16). To date, no comprehensive bibliometric analysis has been conducted in this field. Therefore, we conducted a bibliometric analysis using literature related to PCOS and depression to describe the current status, trends, hotspots, and leading contributors in this field.

## 2 Materials and methods

### 2.1 Data collection

Data were retrieved from the Web of Science Core Collection database (https://www.webofscience.com/wos/woscc/basic-search) on May 27, 2024. The search formula was [TS = (polycystic ovary syndrome) OR (PCOS)] AND [TS = (depression) OR (depressive disorder)] AND LA = (English). The document types were chosen as “article” and “review article.” With the timespan covering January 1, 1993, to May 27, 2024, 777 records were selected and exported as plain text files with full records and cited references.

### 2.2 Data analysis

Bibliometric analyses were performed using WoS and CiteSpace (version 6.1.R6), produced by Professor Chaomei Chen. The time cited, publications over time, categories, citation topic meso, citation topic micro, country, institution, journals, high-cited publications, and funding agencies are available in WoS. Thus, Microsoft Office Excel was used to visualize the WoS results as bar graphs. The keyword co-occurrence, reference co-citation, and country or institution collaboration were analyzed using the CiteSpace software, with the time slicing set as 1 year per slice from January 1993 to May 2024, link scope as within slices, link strength as cosine, and selection criteria as the g-index (*k* = 25). To visualize co-occurrence or co-citation networks, nodes of the same color were clustered into one group with closely related units.

## 3 Results

### 3.1 General analyses

**Figure 1:**
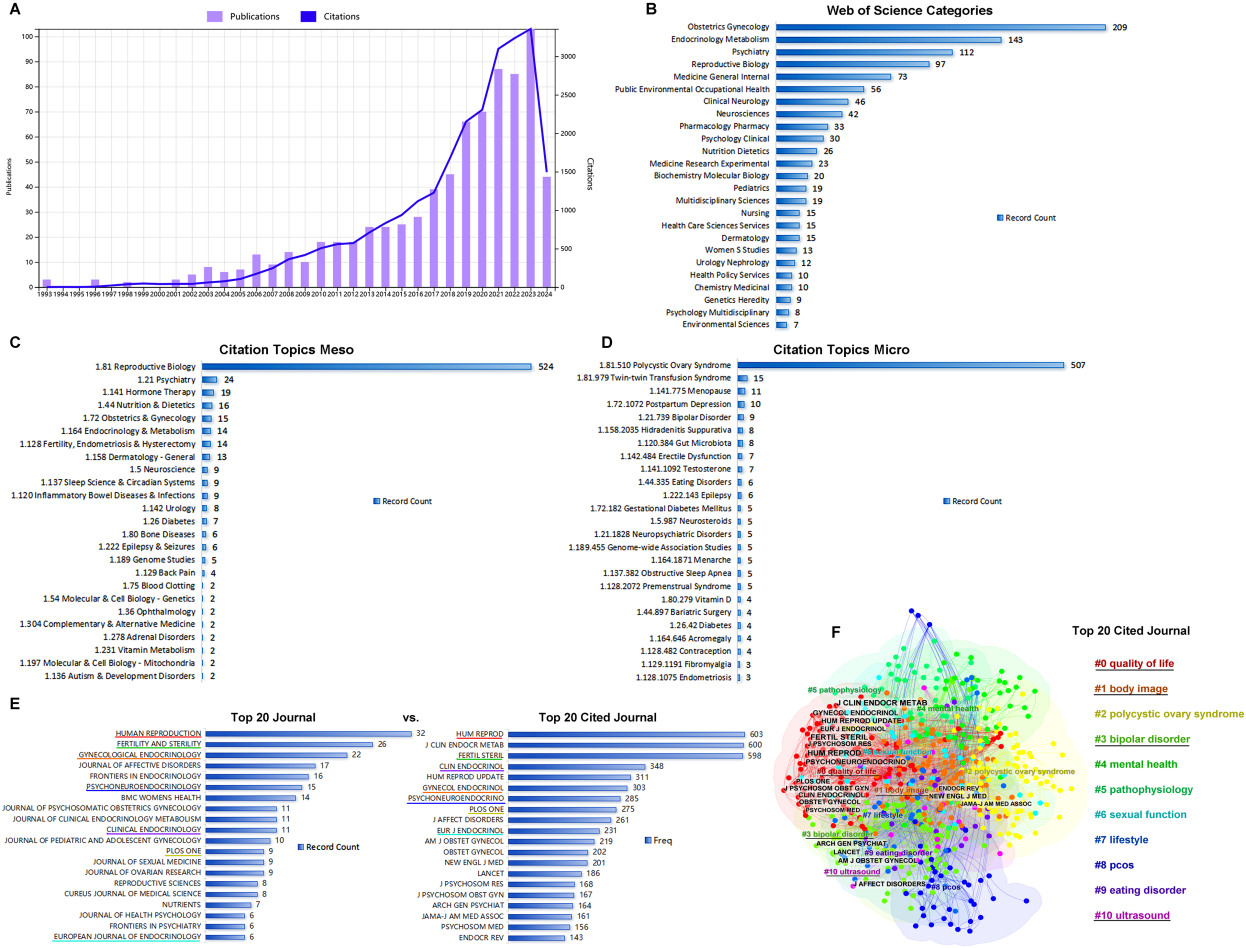
General analysis of publication. **(A)** The times cited and publications over time from 1993.01.01 to 2024.05.27. **(B)** The top 20 categories of publications. **(C)** The top 20 citation topics meso of publications. **(D)** The top20 citation topics micro of publications. **(E)** The top 20 journals vs. top 20 cited journals. **(F)** The clusters of the top 20 cited journals.

General analyses of publications, citations, categories, and journals were performed using WoS. During the search period, a total of 777 publications, including 615 articles (79%) and 162 reviews (21%), were retrieved from the WOS Core Collection. After the first publication in 1993 (Figure 1A), publications and citations were rare before 2003. Since 2003, the number of publications and citations steadily increased, with the sum of publications reaching 777 and the corresponding citations reaching 28,515 without self-citations. The top 20 high cited publications and top 20 co-cited references were listed in Table 1 and Table 2 respectively.

**Table 1:** Citation analysis: the top 20 high cited publications from 1993 to 2024.

No.	Title	Authors	Country	Journal	Year	DOI	Total Citations	Average per year
1	Lack of Exercise Is a Major Cause of Chronic Diseases	Booth FW, et al.	USA	Compr Physiol.	2012	10.1002/cphy.c110025	1,412	108.62
2	Polycystic ovary syndrome: a complex condition with psychological, reproductive and metabolic manifestations that impacts on health across the lifespan	Teede H, et al.	Australia	BMC Med.	2010	10.1186/1741-7015-8-41	819	54.6
3	Epidemiology, diagnosis, and management of polycystic ovary syndrome	Sirmans SM, et al.	USA	Clin Epidemiol.	2014	10.2147/CLEP.S37559	596	54.18
4	The regulation of adipose tissue distribution in humans	Björntorp P.	Sweden	Int J Obes Relat Metab Disord.	1996	No record	514	17.72
5	Sleep apnea is a manifestation of the metabolic syndrome	Vgontzas AN, et al.	USA	Sleep Med Rev.	2005	10.1016/j.smrv.2005.01.006	377	18.85
6	Psychiatric and medical comorbidities of bipolar disorder	Krishnan KR.	USA	Psychosom Med.	2005	10.1097/01.psy.0000151489.36347.18	348	17.4
7	High prevalence of moderate and severe depressive and anxiety symptoms in polycystic ovary syndrome: a systematic review and meta-analysis	Cooney LG, et al.	USA	Hum Reprod.	2017	10.1093/humrep/dex044	290	36.25
8	Quality of life, psychosocial well-being, and sexual satisfaction in women with polycystic ovary syndrome	Elsenbruch S, et al.	Germany	J Clin Endocrinol Metab.	2003	10.1210/jc.2003-030562	267	12.14
9	Large-scale genome-wide meta-analysis of polycystic ovary syndrome suggests shared genetic architecture for different diagnosis criteria	Day F, et al.	UK	PLoS Genet.	2018	10.1371/journal.pgen.1007813	262	37.43
10	Clinical and psychological correlates of quality-of-life in polycystic ovary syndrome	Hahn S, et al.	Germany	Eur J Endocrinol.	2005	10.1530/eje.1.02024	250	12.5
11	Quality of life and psychological well being in polycystic ovary syndrome	Barnard L, et al.	UK	Hum Reprod.	2007	10.1093/humrep/dem108	249	13.83
12	The Potential Implications of a PCOS Diagnosis on a Woman's Long-Term Health Using Data Linkage	Hart R, et al.	Australia	J Clin Endocrinol Metab.	2015	10.1210/jc.2014-3886	235	23.5
13	Increased risk of depressive disorders in women with polycystic ovary syndrome	Hollinrake E, et al.	USA	Fertil Steril.	2007	10.1016/j.fertnstert.2006.11.039	216	12
14	Sexual dysfunction in men and women with endocrine disorders	Bhasin S, et al.	USA	Lancet.	2007	10.1016/S0140-6736 (07)60280-3	202	11.22
15	Anxiety and depression in polycystic ovary syndrome: a systematic review and meta-analysis	Barry JA, et al.	UK	Hum Reprod.	2011	10.1093/humrep/der197	200	14.29
16	Canadian Network for Mood and Anxiety Treatments (CANMAT) guidelines for the management of patients with bipolar disorder: update 2007	Yatham LN, et al.	Canada	Bipolar Disord.	2006	10.1111/j.1399-5618.2006.00432.x	191	10.05
17	Obesity-related sleepiness and fatigue - The role of the stress system and cytokines	Vgontzas AN, et al.	USA	Ann N Y Acad Sci.	2006	10.1196/annals.1367.023	155	8.16
18	Depression and body image among women with polycystic ovary syndrome	Himelein MJ, et al.	USA	J Health Psychol.	2006	10.1177/1359105306065021	153	8.05
19	QUALITY-OF-LIFE OF HIRSUTE WOMEN	Sonino N, et al.	Italy	Postgrad Med J.	1993	10.1136/pgmj.69.809.186	149	4.66
20	Polycystic ovary syndrome and mental health: A review	Himelein MJ, et al.	USA	Obstet Gynecol Surv.	2006	10.1097/01.ogx.0000243772.33357.84	144	7.58

**Table 2:** Co-citation analysis: the clustered top 20 co-cited references from 1993 to 2024.

Cluster	Title	Author	Journal	Year	DOI	Freq
0	Anxiety and depression in polycystic ovary syndrome: a systematic review and meta-analysis	Barry JA, et al.	Hum Reprod.	2011	10.1093/humrep/der197	30
	Anxiety and depression symptoms in women with polycystic ovary syndrome compared with controls matched for body mass index	Jedel E, et al.	Hum Reprod.	2010	10.1093/humrep/dep384	29
1	High prevalence of moderate and severe depressive and anxiety symptoms in polycystic ovary syndrome: a systematic review and meta-analysis	Cooney LG, et al.	Hum Reprod.	2017	10.1093/humrep/dex044	92
	Androgen Excess- Polycystic Ovary Syndrome Society: position statement on depression, anxiety, quality of life, and eating disorders in polycystic ovary syndrome	Dokras A, et al.	Fertil Steril.	2018	10.1016/j.fertnstert.2018.01.038	53
	The prevalence and phenotypic features of polycystic ovary syndrome: a systematic review and meta-analysis	Bozdag G, et al.	Hum Reprod.	2016	10.1093/humrep/dew218	42
	Polycystic ovary syndrome and psychiatric disorders: Co-morbidity and heritability in a nationwide Swedish cohort	Cesta CE, et al.	Psychoneuroendocrinology.	2016	10.1016/j.psyneuen.2016.08.005	32
	Depression and Anxiety in Polycystic Ovary Syndrome: Etiology and Treatment	Cooney LG, et al.	Curr Psychiatry Rep.	2017	10.1007/s11920-017-0834-2	29
	Weight Loss and Lowering Androgens Predict Improvements in Health-Related Quality of Life in Women With PCOS	Dokras A. et al.	J Clin Endocrinol Metab.	2016	10.1210/jc.2016-1896	25
2	Anxiety, Depression, and Quality of Life in Women with Polycystic Ovarian Syndrome	Chaudhari AP, et al.	Indian J Psychol Med.	2018	10.4103/IJPSYM.IJPSYM_561_17	33
4	Recommendations from the international evidence-based guideline for the assessment and management of polycystic ovary syndrome	Teede HJ, et al.	J Clin Endocrinol Metab.	2018	10.1111/cen.13795	45
	Recommendations from the international evidence-based guideline for the assessment and management of polycystic ovary syndrome	Teede HJ, et al.	Hum Reprod.	2018	10.1093/humrep/dey256	36
	Psychiatric disorders in women with polycystic ovary syndrome: a systematic review and meta-analysis	Brutocao C, et al.	Endocrine.	2018	10.1007/s12020-018-1692-3	34
	Recommendations from the international evidence-based guideline for the assessment and management of polycystic ovary syndrome	Teede HJ, et al.	Fertil Steril.	2018	10.1016/j.fertnstert.2018.05.004	32
	Depression, anxiety and perceived stress in women with and without PCOS: a community-based study	Damone AL, et al.	Psychol Med.	2019	10.1017/S0033291718002076	30
5	Women with polycystic ovary syndrome are often depressed or anxious -: A case control study	Månsson M, et al.	Psychoneuroendocrinology.	2008	10.1016/j.psyneuen.2008.06.003	34
	Increased risk of depressive disorders in women with polycystic ovary syndrome	Hollinrake E, et al.	Fertil Steril.	2007	10.1016/j.fertnstert.2006.11.039	29
	Quality of life and psychological well being in polycystic ovary syndrome	Barnard L, et al.	Hum Reprod.	2007	10.1093/humrep/dem108	28
	Risk of depression and other mental health disorders in women with polycystic ovary syndrome: a longitudinal study	Kerchner A, et al.	Fertil Steril.	2009	10.1016/j.fertnstert.2007.11.022	27
	Prevalence and implications of anxiety in polycystic ovary syndrome: results of an internet-based survey in Germany	Benson S, et al.	Hum Reprod.	2009	10.1093/humrep/dep031	26
7	Polycystic ovary syndrome: definition, aetiology, diagnosis and treatment	Escobar-Morreale HF.	Nat Rev Endocrinol.	2018	10.1038/nrendo.2018.24	29

Publications were distributed into categories, including obstetrics/gynecology (27%), endocrinology metabolism (18%), psychiatry (14%), reproductive biology (13%), and general internal medicine (9%) (Figure 1B). Differing from WoS subject categories, which were manually defined by journal editors to classify the publications, the citation topics were algorithmically classified on the basis of the citation clusters. Citation meso-topics and citation micro-topics were at two levels of the hierarchical classification system, and citation micro-topics were subdivided into the hierarchical tree of citation meso-topics. As shown in Figures 1C,D, 68% of the publications were about reproductive biology, 97% of which were about PCOS. In Figure 1E, *Human Reproduction*, *Fertility and Sterility, and Gynecological Endocrinology* were shown as the top three journals that published the achievements in this area. Comparing the top 20 journals to the top 20 cited journals, we found that *Human Reproduction*, *Fertility and Sterility*, *Gynecological Endocrinology*, *Psychoneuroendocrinology*, *Clinical Endocrinology*, and *European Journal of Endocrinology* were listed among both the top 20 cited journals and the top 20 journals. Using CiteSpace software, the top 20 cited journals were identified and clustered into four groups including #0 quality of life, #1 body image, #3 bipolar disorder, and #10 ultrasound (Figure 1F); cluster #0 contained 13 cited journals among the top 20 (Figure 1F).

### 3.2 Keywords co-occurrence and hotspots

**Figure 2:**
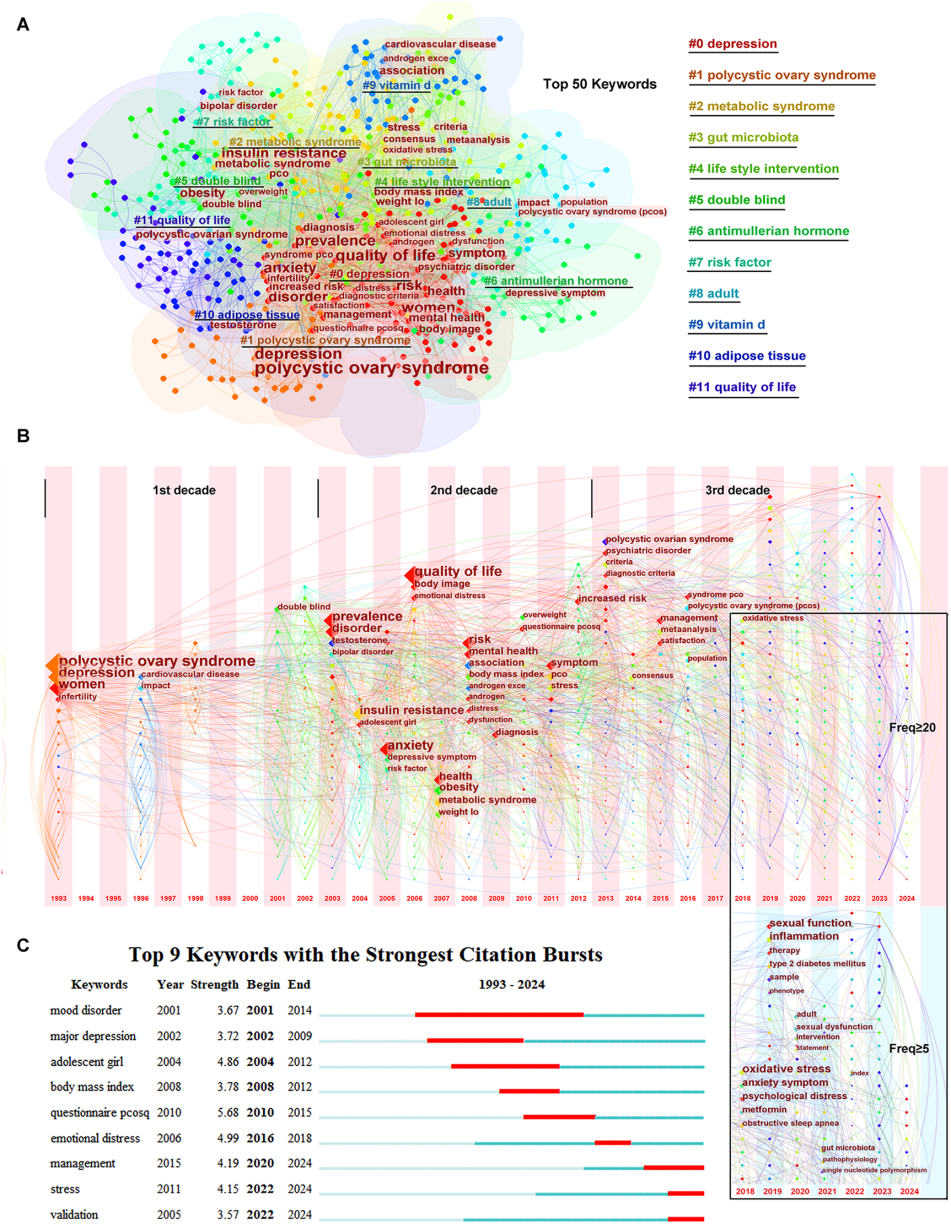
Keywords co-occurrence and hotspots. **(A)** The clusters of the top 50 keywords. **(B)** The timezone of the keywords. **(C)** The citation bursts of the keywords.

Using CiteSpace software, the top 50 keywords were identified from 688 keywords with a frequency ≥20. As shown in Figure 2A, the top 50 keywords were clustered into 12 groups: #0 depression, #1 polycystic ovary syndrome, #2 metabolic syndrome, #3 gut microbiota, #4 lifestyle intervention, #5 double-blind, #6 anti-Müllerian hormone, #7 risk factor, #8 adult, #9 vitamin D, #10 adipose tissue, and #11 quality of life, with half of the top 50 keywords gathered in cluster #0. In these clusters, many keywords, e.g., “insulin resistance,” “obesity,” “androgen excess,” “infertility,” “stress,” “psychiatric disorder,” “metabolic syndrome,” “cardiovascular disease,” “prevalence,” “diagnostic criteria,” “quality of life,” “lifestyle management,” “questionnaire pcosq,” “weight loss,” played vital roles.

The time zone of keywords related to PCOS and depression are shown in Figure 2B. In the time zone network, the selected keyword was presented in the year when it appeared, and the lines between the nodes indicated the connection between the keywords. As shown in Figure 2B, the time zone can be divided into three periods: 1993–2002, 2003–2012, and 2013 until the completion of this research. In the first period (1993–2002), there were few keywords that were mainly limited in “polycystic ovary syndrome,” “depression,” and “women.” However, in the second period (2003–2012), many keywords appeared, and almost all keywords related to pathogenesis were gathered during this period. In the third period (2013 until the completion of this research), considering that the latest published keywords might have been underestimated, the frequency of keywords was set to ≥5 in the last 5 years, differing from the frequency of ≥20 from 1993 to 2017. New diagnostic criteria and a consensus were pursued at the beginning of the third period, and management to improve life satisfaction was emphasized. In the recent 5 years, new keywords such as “oxidative stress,” “inflammation,” “obstructive sleep apnea,” “gut microbiota,” and “single nucleotide polymorphism” emerged and revealed the hotspots in this field.

As shown in Figure 2C, keywords or studies focused on “mood disorder”/“major depression” or “adolescent girl” or “body mass index” years before, while the citation bursts were displayed as “management,” “stress,” and “validation” in recent years.

### 3.3 Co-cited references and authors

**Figure 3:**
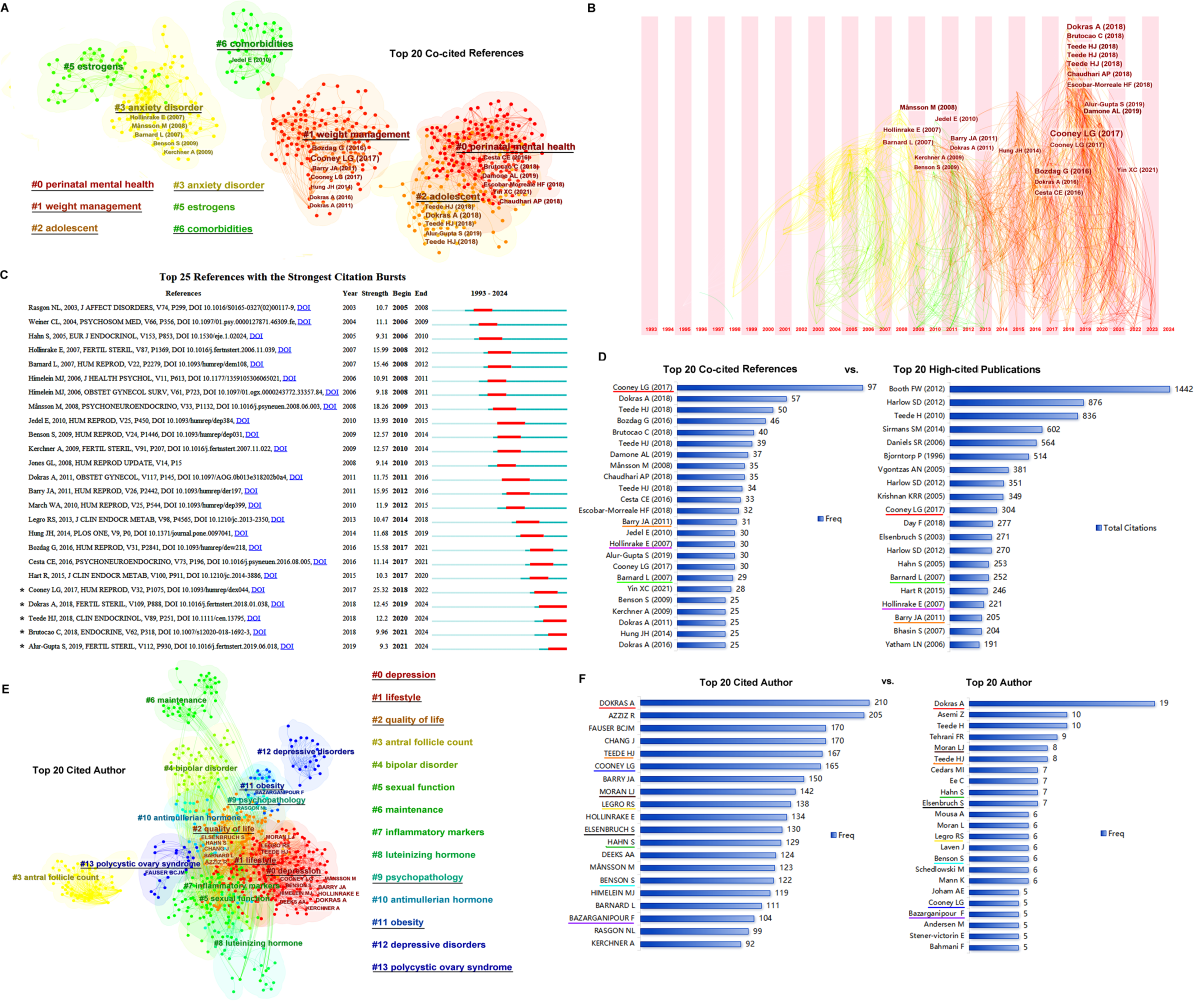
References co-citation and authors. **(A)** The clusters of the top 20 references. **(B)** The timezone of the top 20 references. **(C)** The citation bursts of the references. **(D)** The top 20 co-cited references vs. top 20 high-cited publications. The same papers in the two groups are underlined with the same color, and four papers are presented in each of the top 20 groups. **(E)** The clusters of the top 20 cited authors. **(F)** The top 20 cited authors vs. top 20 authors. The same persons in the cited author list and author list are underlined in the same color.

The top 20 co-cited references, with their frequency of ≥25, were clustered into five groups including #0 perinatal mental health, #1 weight management, #2 adolescent, #3 anxiety disorder, and #6 comorbidities (Figure 3A). In the time zone of Figure 3B, most of the co-cited references in clusters #0, #1, and #2 were published in the third period (2013 until the completion of this research), while those in clusters #3 and #6 were published in the second period (2003–2012). As shown in Figure 3C, studies by Alur-Gupta (2019 cluster #2), Brutocao (2018 cluster #0), Teede (2018 cluster #2), Dokras (2018 cluster #2), and Cooney (2017 cluster #1), among the top 20, were the strongest citation bursts in recent years. The top 20 co-cited references and the top 20 most highly cited publications are compared in Figure 3D.

The authors and cited authors were analyzed using CiteSpace software. As shown in Figure 3E, the top 20 cited authors were clustered into six groups: #0, depression; #1, #2 quality of life, #9 psychopathology, #11 obesity, and #13 polycystic ovary syndrome. The most cited authors were gathered into clusters #0, #1, and #2. The top 20 cited authors and top 20 authors are listed in Figure 3F; a comparison was made between the authors and cited authors, helped identify the most important scientists in this field.

### 3.4 Countries and institutions

**Figure 4:**
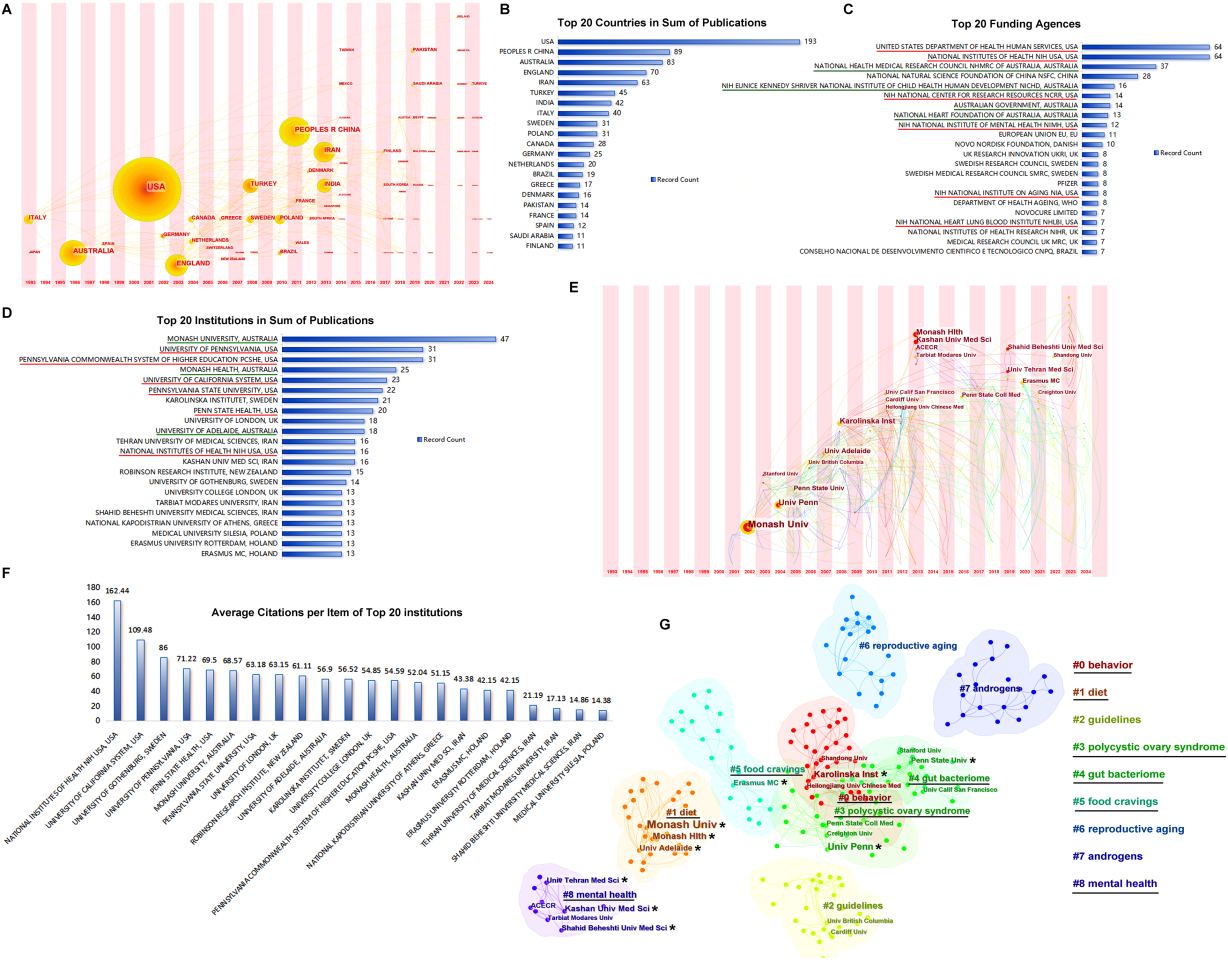
Countries and institutions. **(A)** The timezone of publications by country. **(B)** The top 20 countries in sum of publications. **(C)** The top 20 funding agencies. **(D)** The top 20 institutions in sum of publications. **(E)** The timezone of publications by institution. **(F)** Average citation per item of the top 20 institutions. **(G)** The clusters of publications by institutions.

Over the past three decades, 74 countries have launched publications in this research area (Figure 4A). The United States and Australia, with greater influence (larger dots in the time zone of Figure 4A), were in the first group of counties to start research among the countries participating in these studies. As shown in Figure 4B, the United States took the lead position in the sum of publications, followed by the Peoples' Republic of China, Australia, England, and Iran. The United States and Australia were financially supported by a large number of funding agencies (Figure 4C), of which six funding agencies of the top 20 underlined in red were from the United States and four agencies with green underlines were from Australia.

The top 20 institutions in sum of publications are listed in Figure 4D; institutions in the United States and Australia occupy the front rows. Institutions from Sweden, the United Kingdom, and Iran also assumed important positions. The Monash University in Australia and University of Pennsylvania in the United States were the earliest participants with the largest achievements in these studies (Figure 4E). The average citations per item of the top 20 institutions are shown in Figure 4F. The top institutions from the United States, i.e., the National Institutes of Health (NIH) and University of California System, had much higher average citations than the others. The top 20 institutions marked with stars were clustered into six groups: #0 behavior, #1 diet, #3 polycystic ovary syndrome, #4 gut bacteriome, #5 food cravings, and #8 mental health, as shown in Figure 4G.

## 4 Discussion

The definition of PCOS had been debated for a long time until the expert conference on PCOS sponsored by the National Institute of Child Health and Human Disease (NICHD) of the NIH in 1990. The NIH/NICHD criteria for PCOS in 1990 were as follows: (i) hyperandrogenism and/or hyperandrogenemia, (ii) oligo-ovulation, and (iii) the exclusion of other known disorders. The first study on the association between PCOS and depression was published in 1993. However, publications and citations related to PCOS and depression remained rare in the following decade. The second decade in this field began in 2003 and was influenced by another expert conference sponsored by the European Society for Human Reproduction and Embryology and the American Society for Reproductive Medicine in Rotterdam. The diagnostic criteria for PCOS in 2003, known as the Rotterdam criteria, were recommended as follows: (i) clinical and/or biochemical signs of hyperandrogenism, (ii) oligo- and/or anovulation, and (iii) polycystic ovaries on ultrasonography, which expanded the NIH/NICHD criteria in 1990 and have been widely used until now. Coincidentally, from 2003, the publications and citations related to PCOS and depression grew rapidly and entered a booming period to date.

The achievements in this field are mainly categorized into “obstetrics/gynecology,” “endocrinology metabolism,” “psychiatry,” and “reproductive biology.” Moreover, most of these researches were further classified algorithmically into “reproductive biology” and then subdivided into “polycystic ovary syndrome.” These classifications indicate that PCOS was considered the primary disease in most of these studies, and research on depression has focused on its associations or connections to PCOS. These achievements have been published mainly in professional journals such as *Human Reproduction*, *Fertility*, and *Gynecological Endocrinology*. Furthermore, these professional journals were listed first among the cited journals and clustered into “quality of life,” which indicates that this topic received special attention by professional journals for gynecologists, endocrinologists, or reproductive endocrinologists and that research to improve the quality of life in women with PCOS was particularly emphasized.

The top 50 keywords were clustered to explore the research trends in this field. We summarized the themes of these clusters as follows: (i) the possible mechanisms underlying the increased prevalence of depression in women with PCOS included IR, obesity, androgen excess, infertility, stress, vitamin D, and oxidative stress, inflammation, and gut microbiota; (ii) the comorbidity of PCOS shared links with depressive syndrome including type 2 diabetes mellitus, cardiovascular disease, metabolic syndrome, and other psychiatric disorders, e.g., anxiety and bipolar disorder; (iii) variables related to treatments in women with PCOS and depression, e.g., polycystic ovary syndrome health-related quality of life questionnaire, lifestyle intervention, weight loss, and metformin administration; and (iv) methodology-related meta-analysis and single nucleotide polymorphism.

As mentioned above, the time zones of these studies were divided into three periods. In the first period (1993–2002), the studies related to depression in women with PCOS were rare. In the second period (2003–2012), starting with the Rotterdam criteria in 2003, achievements thrived in this field, and most keywords related to prevalence, pathogenesis, and treatment appeared. In the second period, the prevalence of depression in women with PCOS was estimated using multiple cross-sectional studies from various regions (17–23). Furthermore, many large longitudinal population-based studies have verified the high prevalence of depression in patients (24–27). The potential mechanisms underlying PCOS and depression were explored, and the possible associations among PCOS, depression, stress, HPA axis, obesity, IR, androgen excess, infertility, and inflammation were discussed in depth. Moreover, treatments for PCOS and related depression focused on improving the quality of life of patients, and were evaluated in several randomized controlled trials in the second period (28–30). Lifestyle intervention was considered the first-line treatment, including weight loss, exercise, behavioral management, and psychological interventions, e.g., cognitive behavioral therapy. Pharmacological treatments were prescribed, including oral contraceptive pills used to regulate menses and treat hirsutism; insulin sensitizers, e.g., metformin used in the management of IR and metabolic risk factors; and psychiatric medications, e.g., selective serotonin reuptake inhibitors used to treat depressive syndrome (8–10, 31).

Following the flourishing period, the third period (2013 until the completion of this research) started with the American Endocrine Society (AES) launching new guidelines for the diagnosis and treatment of PCOS in 2013. In those guidelines, the 2003 Rotterdam criteria were suggested for diagnosing PCOS. Thus, the diagnosis of PCOS in adolescents and menopausal women was specified in this guideline, and treatments for PCOS were evaluated (32). Following the consensus of the 2013 AES guidelines, research has focused on improving the quality of life of women with PCOS and depression. In 2018, an evidence-based guideline was updated in Australia, with changes mainly in the refinement of diagnostic criteria and an increasing focus on quality of life and fertility management (1). In the 2018 guideline, it was recommended that depressive syndrome be routinely screened for in women with PCOS, including adolescents, at diagnosis. Moreover, for the first time, obstructive sleep apnea was recommended for screening in PCOS patients with related syndromes.

After the 2018 guideline was launched, new hotspots appeared, such as “oxidative stress,” “inflammation,” “obstructive sleep apnea,” “gut microbiota,” and “single nucleotide polymorphism.” “Oxidative stress” and “inflammation” are vital causes of PCOS and depression (33, 34). Failure to maintain redox homeostasis results in the generation of pro-inflammatory mediators and leads to the pathogenesis of PCOS and depression. Considering oxidative stress and inflammatory cytokines as markers, multiple clinical trials were performed from 2018 until now to verify the positive effects of nutrient supplementation including specific vitamins (B-12, inositols, folate, and vitamins D, E, and K), vitamin-like nutrients (bioflavonoids and α-lipoic acid), minerals (calcium, zinc, selenium, and chromium picolinate), and other formulations (melatonin, ω-3 fatty acids, probiotics, and cinnamon) in women with PCOS and depression (35–46).

“Obstructive sleep apnea” was found to be closely associated with PCOS, especially in PCOS patients with depressive syndrome. The underlying mechanisms may be related to depressive syndrome, obesity, hyperandrogenemia, IR, and an abnormal HPA axis in PCOS but are not yet clear. The relationship between PCOS, depressive syndrome, and obstructive sleep apnea might be bidirectional. Given that PCOS and obstructive sleep apnea are risk factors for cardiometabolic health and type 2 diabetes, the diagnosis and management of obstructive sleep apnea should be beneficial for the immediate quality of life and long-term health of women with PCOS and depression (47–52).

“Gut microbiota” appeared as a hotspot in this field in 2021. Changes in the overall composition, including decreases in alpha and beta diversities of the gut microbiota, are associated with PCOS, while compositional changes in the gut microbiota community, e.g., reduced diversity along with a decrease in short-chain fatty acid synthesis, are found in patients (53–57). Furthermore, in a population-based cohort study, Lee demonstrated that the gut microbiota was associated with depression irrespective of the PCOS status and that PCOS further modulated the connection between the gut microbiota and depression (58). The underlying mechanisms related to the gut microbiota and pathogenesis of PCOS with depressive syndrome remain unclear. However, gut microbiota replacement therapeutic strategies such as fecal microbiota transplantation, prebiotic therapy, probiotic therapy, synbiotic therapy, and psychobiotic therapy are optimized for their efficacy (53, 55).

PCOS is a complex polygenic disorder caused by the interaction between a vast array of genetic and environmental factors. PCOS and depression-associated “single nucleotide polymorphisms” were hotspots in 2021. Whether there are shared genetic bases between PCOS and depression has been discussed in multiple studies, but different results have been reported in various researches (59–64). Thus, further studies are required to address this issue.

On the basis of the new trends and hotspots in this field, we determined that as one of the most common disorders threatening women's health across their lifespan, the pathogenesis of PCOS remains uncertain due to heterogeneity. However, in the fast-evolving field, new avenues of research on pathogenesis are rapidly increasing to fill the gaps, and corresponding treatment strategies are subsequently emerging.

References related to PCOS and depression were clustered to identify the important studies and their internal connections. Recently, studies related to perinatal mental health, weight management, and adolescence have received increasing attention. Studies by Cooney (2017), Dokras (2018), Brutocao (2018), Teede (2018), and Alur-Gupta (2019) are considered the most important references and strongest citation bursts in recent years. Cooney (2017) performed a meta-analysis and reported that compared to women without PCOS, those with PCOS have significantly increased odds of moderate and severe depressive and anxiety symptoms, independent of obesity, and that the symptoms are weakly associated with age, body mass index, elevated testosterone, hirsutism, and IR (5). In another meta-analysis, Dokras (2018) indicated that women with PCOS have an increased prevalence of depression and higher odds of moderate and severe depressive symptoms than controls. The prevalence of eating disorders has also increased among women with PCOS. Obesity, hyperandrogenism, and fertility are weakly associated with these symptoms (65). Brutocao (2018) also performed a meta-analysis and showed that PCOS was associated with an increased risk of depression, anxiety, bipolar disorder, and obsessive-compulsive disorder (17). Teede (2018) summarized the international evidence-based guideline to recommend that depressive syndrome should be routinely screened in all patients with PCOS at diagnosis (1). Alur-Gupta (2019) conducted a cross-sectional study and demonstrated that different aspects of body image distress either fully or partially mediated the association between PCOS and depression scores in women with PCOS (18). Most of the top authors such as Dokras, Cooney, Alur-Gupta, and Legro from the United States and Teede and Moran from Australia, who devoted years and are still working in this field, focused their research on lifestyle interventions and improving the quality of life in women with PCOS and depression.

Geographic analysis revealed that in the past three decades, 74 countries have participated in research in this area. Because local researchers started earlier and achieved more in the studies, the United States and Australia occupy the leading positions not only in the sum of publications and average citations per item, but also in the quantity of financial support from funding agencies and top institutions in this area. The University of Pennsylvania in the United States and Monash University in Australia are the most accomplished institutions in this research area.

## 5 Conclusions

Here, we described the trends and hotspots of research in women with PCOS and depression to attract the attention of more researchers to this topic. Research on depressive syndrome in women with PCOS began in 1993 and has been conducted for more than three decades. Much attention has been focused on the confounding disorders that threaten women's health across their lifespan. Since the establishment of the Rotterdam criteria in 2003, research on the etiology, pathogenesis, and treatment of PCOS with depressive syndrome has entered a boom. Although achievements have flourished since 2003, the exact pathogenesis of PCOS remains uncertain owing to its heterogeneity. New research on validation, pathogenesis, and treatment is rapidly increasing to fill the gaps in this area and push forward the ultimate goal of improving the quality of life of women with PCOS and depression.

## 6 Limitations

This study is the first exploration of the bibliometric analysis on researches from 1993 till now in women with PCOS and depression. However, some limitations should be noted. First, although the data in this study was extracted from WoSCC which is the world's leading citation database, other databases such as Scopus or Google Scholar may contain publications various from WoSCC. Second, only English publications were included in the analysis. Finally, some of the high-quality but new published articles might been missed due to the low citation frequency. Therefore, new published articles, non-English articles as well as articles incorporated in other databases were underestimated in this study which may cause some biases to the results in the specified time period.

## Data Availability

The original contributions presented in the study are included in the article/Supplementary Material, further inquiries can be directed to the corresponding author.
